# Exposure to mobile telecommunication networks assessed using personal dosimetry and well-being in children and adolescents: the German MobilEe-study

**DOI:** 10.1186/1476-069X-7-54

**Published:** 2008-11-04

**Authors:** Silke Thomas, Anja Kühnlein, Sabine Heinrich, Georg Praml, Rüdiger von Kries, Katja Radon

**Affiliations:** 1 Unit for Occupational and Environmental Epidemiology and Net Teaching, Institute and Outpatient Clinic for Occupational-, Social- and Environmental Medicine, Ludwig-Maximilians-University Munich, Ziemssenstr. 1, 80336 Munich, Germany; 2 Institute for Social Paediatrics and Adolescent Medicine, Ludwig-Maximilians-University, Heiglhofstr. 63, 80377 Munich, Germany

## Abstract

**Background:**

Despite the increase of mobile phone use in the last decade and the growing concern whether mobile telecommunication networks adversely affect health and well-being, only few studies have been published that focussed on children and adolescents. Especially children and adolescents are important in the discussion of adverse health effects because of their possibly higher vulnerability to radio frequency electromagnetic fields.

**Methods:**

We investigated a possible association between exposure to mobile telecommunication networks and well-being in children and adolescents using personal dosimetry. A population-based sample of 1.498 children and 1.524 adolescents was assembled for the study (response 52%). Participants were randomly selected from the population registries of four Bavarian (South of Germany) cities and towns with different population sizes. During a Computer Assisted Personal Interview data on participants' well-being, socio-demographic characteristics and potential confounder were collected. Acute symptoms were assessed three times during the study day (morning, noon, evening).

Using a dosimeter (ESM-140 Maschek Electronics), we obtained an exposure profile over 24 hours for three mobile phone frequency ranges (measurement interval 1 second, limit of determination 0.05 V/m) for each of the participants. Exposure levels over waking hours were summed up and expressed as mean percentage of the ICNIRP (International Commission on Non-Ionizing Radiation Protection) reference level.

**Results:**

In comparison to non-participants, parents and adolescents with a higher level of education who possessed a mobile phone and were interested in the topic of possible adverse health effects caused by mobile telecommunication network frequencies were more willing to participate in the study. The median exposure to radio frequency electromagnetic fields of children and adolescents was 0.18% and 0.19% of the ICNIRP reference level respectively.

**Conclusion:**

In comparison to previous studies this is one of the first to assess the individual level of exposure to mobile telecommunication networks using personal dosimetry, enabling objective assessment of exposure from all sources and longer measurement periods. In total, personal dosimetry was proofed to be a well accepted tool to study exposure to mobile phone frequencies in epidemiologic studies including health effects on children and adolescents.

## Background

The use of mobile communication devices has increased within the last years. At the same time, there is growing public concern that radio frequency electromagnetic fields could cause adverse health effects even at exposure levels far below the reference levels [1]. In a recent study in Germany, 27% of the participants reported concerns about such effects [2].

Mainly people living near to a mobile phone base station are concerned about potential harmful effects of their radiation [3]. People relating their health problems to base stations or mobile phones often report having unspecific symptoms like headache, restlessness, sleep disturbances, concentration and memory problems, absence of appetite as well as tinnitus [4-6].

In the discussion of possible adverse health effects caused by mobile phones children and adolescents are relatively important, because they are possibly more sensitive to radio frequency electromagnetic fields. Among others, a possible higher vulnerability of children and adolescents is mainly discussed, because of a greater susceptibility of their developing nervous system. Furthermore today's children and adolescents have a higher cumulative exposure during their lifetime as today's adults, because they start their use earlier in life [7-9]. In addition, recent studies indicated higher SAR values for children in comparison to adults [10-12].

Up to now there are only few epidemiologic studies published that investigated a possible association between exposure to mobile telecommunication networks and health outcomes in children and adolescents. The results of two Finnish studies and one Swedish study that investigated the use of mobile phones and the association with health symptoms in adolescents showed that frequent mobile phone use was associated with poor perceived health [13-15]. One main drawback of these studies was that the exposure assessment had to rely on self-reports of the participants.

The same problem underlines most other epidemiologic studies which focus on adults [16,17]. Most of such studies using self-reported exposure and outcome found some association which might be due to a differential misclassification of exposure. This bias is also called "awareness bias" [18].

Hutter and colleagues have attempted to address this problem in their pilot study by measuring exposure to base stations in the participants' bedrooms [3]. However, the issue could not be fully resolved. Electromagnetic emissions of a mobile phone base station vary over time and indoor field strength strongly depends on the station's position, making it difficult to capture the exposure accurately [19]. In addition, bedtime exposure reflects only one part of the overall exposure. Using the same method of exposure assessment, Berg et al. examined in a population based cross-sectional study also possible health effects caused by exposure to mobile phone base stations on adults. The results showed that participants who were concerned about or attributed adverse health effects on mobile phone base stations reported more often health complaints than the remaining participants. No association between measured exposure and health was found [20].

Until recently, there is only one epidemiological study which used personal dosimetry to assess the individual exposure. The aim of the study – also performed by our workgroup-was to investigate a possible association between exposure to mobile telecommunication networks and well-being in adults. The results showed an exposure markedly below the ICNIRP reference levels (highest exposure in Munich: 0.41% of the ICNIRP reference level). No association between exposure and symptoms was found [21].

### Objectives

The objective of this study was to investigate a potential association between exposure to mobile telecommunication networks and well-being in children and adolescents and to assess the level of exposure in a general-population sample of children and adolescents living in Bavaria (Southern Germany).

The current paper presents the methods, descriptive data and the results of a non-response analysis. Separate papers, based on the full MobilEe-study, will address the possible association between exposure to mobile phone frequencies and (1) acute symptoms, (2) chronic symptoms and (3) mental health behaviour.

## Methods

### Study population

The MobilEe-study is a population based cross-sectional study taking place in four Bavarian cities with different population size: Munich (~1.300.000 inhabitants), Augsburg (~260.000 inhabitants), Rosenheim (~60.000 inhabitants) and Landsberg (~28.000 inhabitants) [22]. The participants were randomly selected from the registration offices of these four towns. The following selection criteria were used:

▪ Children between 8–12 years old, adolescents between 13–17 years.

▪ German nationality in order to diminish language difficulties.

▪ If more than one sibling from one household was selected they were excluded to minimise cluster effects.

### Study design and procedure

The field-phase of the study started in February 2006 and was completed in December 2007. Overall, 1.498 children and 1.524 adolescents were included. Written informed consent was obtained of the participants' parents and – if they were older than 14 years – of the adolescents themselves.

Each family of a potential participant got a letter consisting of information about the study, an informed consent form and a short questionnaire. All subjects invited were asked to answer the short questionnaire, irrespective of their participation in the field study. This information was used to assess potential selection bias. In case of the children the parents answered the questionnaire, in case of the adolescents they answered themselves. Non-responders got up to two postal reminders and were contacted by phone up to five times. Missing data on the short questionnaire were assessed in a phone interview.

Those who declared consent were invited to a local study centre, where they completed a Computer Assisted Personal Interview (CAPI) with questions on chronic symptoms and potential confounding variables. The duration of the interview was about 30 minutes. In case of the children one parent was also invited to complete an interview. Besides the same questions as their children the parents were also asked about their environmental worries and socioeconomic characteristics (e.g. income, education level). For adolescents this information was obtained directly from the teenagers.

**Figure 1:**
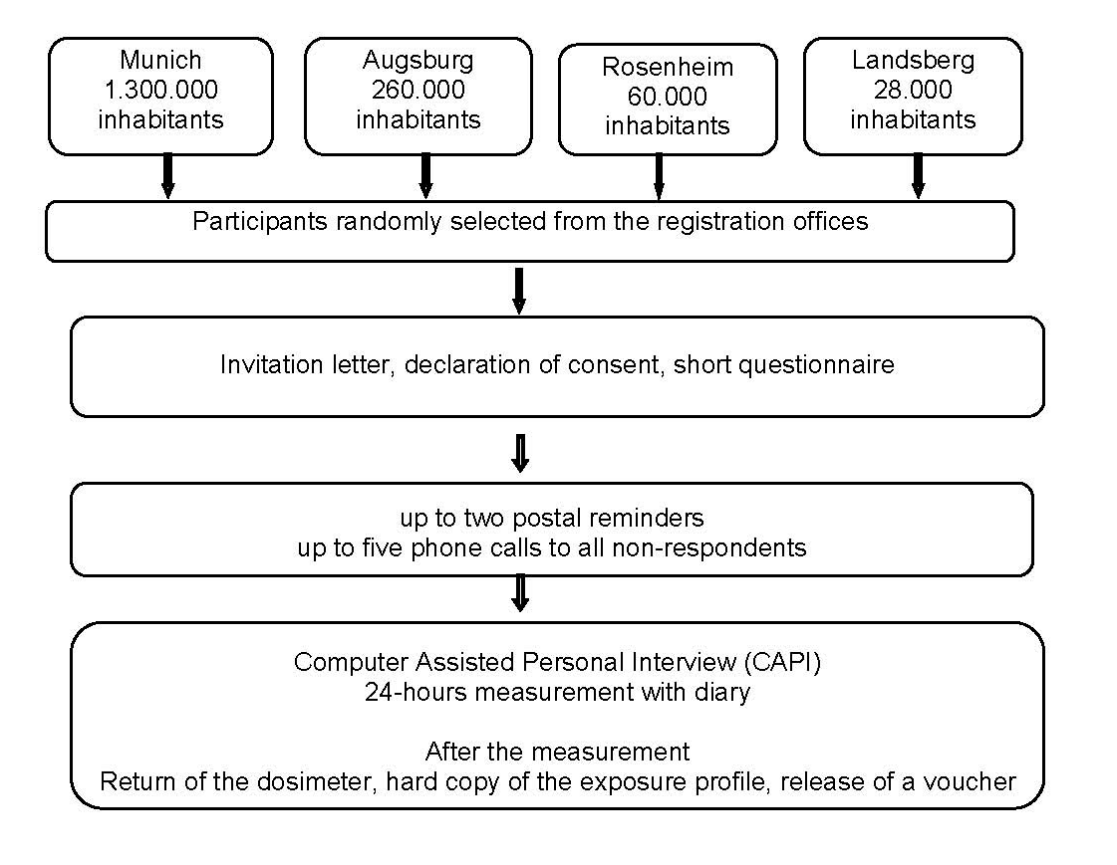
Study procedure of the MobilEe-study.

After the interview the ESM-140 dosimeter was handed out to the children and adolescents for a 24 hours measurement. During this measurement, the participants were asked to fill in a diary recording acute symptoms at noon and in the evening before bedtime as well as the frequency of mobile phone calls and DECT phone (Digital Enhanced Cordless Phone) calls in the previous hours. As an incentive, participants obtained a 20-Euro purchase voucher (Figure 1).

After an interview training, all field workers were retrained again during the field phase. Quality controls were done on a weekly base. In addition, some participants were selected at random after the field study and asked by the coordinators whether they felt comfortable.

The study was approved by the Ethics Committee of the Medical Faculty of the Ludwig-Maximilians-University Munich (285/03).

### Questionnaire instruments

The following described questionnaire instruments, that were used in the study, are shown in table 1.

**Table 1:** Questionnaire instruments

**Outcome**	**Measuring instrument**
Acute symptoms	Typical symptoms mentioned in the context of exposure to mobile phone frequencies, answers following the German "Zerssen complaint list" [23]
Chronic symptoms	Following the questions of the HBSC-study (Health Behaviour in School-aged Children) [24]
Mental health	German version of the Strength and Difficulties Questionnaire (SDQ) [25-27]
**Potential confounding variables**	
Sociodemographic data	Questionnaire of the German Health Interview and Examination Survey for Children and Adolescents [28]
Environmental worry	Short form of the environmental worry scale [29,30]
**Exposure**	
Objective exposure	Measurement using personal dosimetry
Subjective exposure	Questionnaire of the annual survey of the infas from 2003 [31]

#### Acute symptoms

The following acute symptoms were assessed three times during the 24 hours measurement using a paper-based diary: headache, irritation, nervousness, dizziness, fatigue and concentration problems.

The diary items were taken from the "Zerssen complaint list" [23] and assessed on a four point Likert scale (heavy, moderate, barely, not at all). The symptoms were considered present if they were reported with an at least barely intensity.

#### Chronic symptoms

Chronic symptoms during the last six months were assessed during the CAPI. The symptoms were taken from the questionnaire of the HBSC-survey (Health behaviour in School-Aged Children) and consisted of the following ones: headache, irritation, nervousness, dizziness, fatigue, fear and sleeping problems [24].

The list was assessed on a five point Likert scale (nearly daily, several times a week, nearly every week, about once a month, seldom or never) during the CAPI. All symptoms were considered present if they occurred at least nearly every week according to self-report.

#### Mental health

The 25 questions concerning mental health behaviours were asked during the CAPI following the German version of the Strength and Difficulties Questionnaire (SDQ). The questionnaire contained 5 scales: emotional symptoms, conduct problems, hyperactivity/inattention, peer relationship problems and prosocial behaviour. Each scale comprised 5 items with three possible forms ("certainly true", "somewhat true", "not true"). The summary score for each scale could range from 0–10 points. The difficulties score was generated by the summation of all four scales except the prosocial scale. The overall score could range from 0–40 points. For each scale as well as for the total difficulties score the participants were classified as "normal", "borderline" or "abnormal". [25-27]

Due to the small numbers of children and adolescents that could be classified as "borderline", we used two groups for the analysis ("normal" and "borderline/abnormal") and those participants who were in the borderline group were classified as "abnormal".

#### Potential confounder

Sociodemographic data, the four study towns and environmental worries taken from the CAPI were considered as potential confounder.

The sociodemographic data included age, sex and level of education as confounder [28]. All variables were dichotomised:

▪ Age group:

8–10 and 11–12 years (children) respectively 13–15 and 16–17 years (adolescents)

▪ Level of education:

at least 12 years of education (parents of the children) respectively at least grammar school ("Gymnasium") (adolescents)

The four study towns Munich, Augsburg, Rosenheim and Landsberg were also considered as potential confounding variables.

#### Environmental worries

Environmental worries were assessed during the CAPI for the parents of the children and for the adolescents themselves with a short form of the environmental worry scale [29,30]. This scale consists of 12 questions about general and specific environmental worries (e.g. noise exposure or general environmental pollution) and was assessed on a four-point Likert scale (fully agree, partly agree, partly disagree, fully disagree). We a priori decided to dichotomise the variable using the median as cut off. This was done because there is no standard strategy to evaluate the environmental worry scale.

#### Self estimated exposure to mobile telecommunication networks (subjective exposure)

Besides the objective assessment of exposure using personal dosimetry participants were asked to estimate their exposure to mobile telecommunication networks. Participants estimated their exposure during the measurement day using a diary ("How many minutes did you use a mobile phone or DECT phone within the last 8 hours?"). In the baseline interview participants were also asked about the distance to the next mobile phone base stations in the living environment ("How many meters is the next mobile phone base station away from your home?"). The questions were taken from the questionnaire of the annual survey of the Institute for applied sciences (infas) and provided different reply categories [31].

These exposure estimates have been used to compare our results to previous studies on possible associations between self-reported exposure to mobile phone frequencies and health [4,16,17,32,33].

#### Exposure assessment using personal dosimetry

Exposure was measured using the personal dosimeter ESM-140 (Maschek Electronics). For 24 hours the dosimeter was placed on the upper arm of the participants opposite to the side which they usually used to hold the mobile phone or DECT phone during phone calls. During the measurement, subjects were asked to perform their routine daily tasks. At night, the participants placed the dosimeter next to their beds. After handing back the dosimeter the local assistants immediately read out the profiles and checked them for completeness and plausibility.

Exposure was assessed every second resulting in 86.400 measurements over 24 hours. The following frequency ranges were covered [17]:

▪ GSM 900 (up and down link [up link: mobile phone; down link: mobile phone base station]; frequency range: 890–960 MHz)

▪ GSM 1800 (up and down link; frequency range: 1710–1880 MHz) including UMTS 2100 (frequency range: 1920–2170 MHz) and DECT (frequency range: 1880–1900 MHz)

▪ WLAN 2400 frequencies (frequency range: 2400–2480 MHz)

For the exposure assessment, a combination of the frequency bands had to be used because the dosimeter has a low selectivity between the up- and down-link channels.

Four dosimeters were selected at random and tested at the Technical Inspection Agency South (TÜV Süd) to confirm the technical data given by the manufacturer and to investigate if the different dosimeters used in the study are comparable. The findings showed a good comparability of the different dosimeters. At a given exposure level of 0.5 V/m the relative standard deviation was between 4% (DECT) and 17% (GSM 1800). Furthermore the findings proved the validity of the technical data provided by the manufacturer. According to the results of these laboratory measurements, the limit of determination was set at 0.05 V/m. To classify the exposure all measured values that were below this limit were replaced previous to the analysis by half of the limit (0.025 V/m). This method is often used in context of environmental epidemiology and the results seem plausible, because all values have to be between 0 and the limit of determination [34].

As a summary of the measured exposure we calculated in a first step the squared field strength, averaged over the waking hours, for the three frequency ranges. This way e.g. the squared field strength Ē^2 ^for GSM 900 has been calculated via the following formula:

$$\begin{array}{lll} {\bar{\text{E}}}_{\text{GSM }900}^{2} & = & ((\Sigma_{\text{waking hours}}{\text{E}}_{\text{GSM }900\text{ uplink}}^{2}(\text{t}))/\text{duration of waking hours})+\\ & = & \left((\Sigma_{\text{waking hours}}{\text{E}}_{\text{GSM }900\text{ downlink}}^{2}(\text{t}))/\text{duration of waking hours}\right) \end{array}$$

The overall simultaneous exposure to multiple frequency fields was calculated by summation on the squared field strengths, averaged over waking hours, which are weighted by the inverse of squared ICNIRP reference level [35]. Extracting the square-root over this sum returned an overall exposure in terms of field strengths percentage of the reference level [21]:

Overall exposure_% _= square-root ((Ē^2^_GSM 900_/limit^2^_900 MHz_) + (Ē^2^_GSM 1800_/limit^2^_1800 MHz_) + (Ē^2^_WLAN_/limit^2^_2400 MHz_)) * 100

#### Level of exposure during bedtime

During night the participants were asked to fix the dosimeter next to their bed on a bottle filled with water as a replacement for the participants' arm. The question came up if the measured values at this fixed position could represent the real exposure.

Therefore, the Technical Inspection Agency South (TÜV Süd) also investigated if the stationary measured bedtime exposure was a valid proxy of night time exposure. It was confirmed in the lab measurements that the measurement results of the dosimeter measurements depend on the direction in the field. So valid measurements of the average exposure can only be obtained if the dosimeter is moved. Due to these results bedtime exposure levels were not considered to be a valid proxy of night time exposure. Thus these levels had to be excluded and only exposure levels during individual waking hours were used.

#### Five day measurements

To verify the representativeness of the 24 hours measurements 54 participants carried the dosimeter for five consecutive days from Monday afternoon to Saturday afternoon.

For the analysis the time between 4.p.m of one day until 4 p.m. of the following day (night time values were excluded) was summed up and exposure during waking hours was used. Exposure was classified into quartiles for each study day. The overall agreement of these quartiles between the weekdays was assessed. As a random misclassification of exposure on neighbouring cells always results in an underestimation of the effect [36], the deviation by at maximum 1 category was also considered.

### Statistical analysis

In the main analysis the potential association between the measured exposure to mobile telecommunication networks and acute and chronic well-being was analysed.

The objective exposure was divided into quartiles. For these analyses, the mean percentage of the ICNIRP reference level during waking hours was used to analyze the association between exposure to mobile phone frequencies and chronic symptoms. The mean percentage of the ICNIRP reference level during morning hours (time when diary was completed in the morning – time when diary was completed at noon) was considered relevant for the relationship between morning exposure and acute symptoms at noon. Finally, the mean percentage of the ICNIRP reference level during afternoon hours (time when diary was completed at noon – time when diary was completed in the evening) was calculated to test the potential association between afternoon exposure and acute symptoms before bedtime.

In a secondary analysis, self-reported data on distance of the home to the next mobile phone base station as well as the data on usage of mobile phones and DECT phones on the study day were used as exposure proxy. Self estimated distance of the home of the participants to the next base station was used as exposure proxy to analyze the association with chronic symptoms. For acute symptoms, diary data on usage of mobile phones and DECT phones during the study day were used as exposure proxy.

In a sensitivity analysis we also evaluated exposure separately for the three frequency bands GSM 900, GSM 1800 (including UMTS and DECT) and WLAN and thus included three variables in the model. In a second sensitivity analysis exposure was considered a binary cut-off (90% percentile).

Chi^2^-tests were used to assess bivariate associations. Multivariate analyses were done using logistic regression models adjusted for age, sex, level of education, environmental worry, frequency of mobile phone use, frequency of DECT phone use, estimated distance to the next mobile phone base station and study place. These potential confounding variables were defined a priori and included in all analyses. Statistical analyses were carried out using SAS (SAS version 9.1; SAS Institute Inc., Cary, NC, USA).

## Results

### Participation

Overall 6.386 children and adolescents were invited, of which 5.870 were eligible for the study. 4.452 persons (76%) answered the short questionnaire and 3.022 children and adolescents (52%) participated in the study. Adolescents (78%) answered the questionnaire more frequent than parents (74%), however parents participated in the study a little more frequent (53%) than adolescents (51%). The readiness to take part in the study was lowest in Augsburg (46%) and highest in Landsberg (59%).

#### Non-response analysis

To analyse a possible bias caused by selective non-participation, we compared the data of the short questionnaire of those who participated in the field study to those who did not. Parents of participating children were more likely mobile phone owners (93% of the participants, 90% of the non-participants; p_Chi2 _0.02). Parents who had a higher level of education (at least 12 years of education) and those who were concerned about mobile phone exposure were more likely to take part in the study. Furthermore participating parents more often knew the distance to the next base station (don't know: 3%) than those who did not want to participate (don't know: 22%).

Participating adolescents were also more likely to have a higher level of education (at least grammar school) and to be more frequent mobile phone owners (91%) than non-participants (87%). Regarding the self estimated distance from home to the next mobile phone base station, non-participating adolescents (17%) were more likely to choose the "don't know" category than those who participated (3%). Furthermore participating adolescents were also more concerned about mobile phone exposure (at least fairly concerned: 12%) than non-participants (at least fairly concerned: 8%) (Table 2).

**Table 2:** Comparison of parents and adolescents who participated in the field study to non- participants (data from the short questionnaire)

**Variable**	**Participants**	**Non-participants**	
	**Prevalence n (%)**	**Prevalence n (%)**	**p-value**
**Parents of the children**	**n = 1477**	**n = 628**	
Sex			0.29
male	359 (24.3)	139 (22.2)	
Level of education			
at least 12 years of education	748 (51.0)	250 (40.4)	<0.0001
Nationality (mother)			<0.0001
German	902 (85.6)	347 (72.0)	
Mobile phone ownership			0.02
	1365 (92.9)	562 (89.8)	
Use of a mobile phone per day			0.16
< 5 minutes	1057 (72.4)	475 (76.4)	
> 5 minutes	404 (27.7)	147 (23.7)	
Self-reported concerns about adverse health effects to EMF			<0.0001
not or little concerned	580 (55.4)	350 (72.5)	
fairly concerned	386 (36.9)	103 (22.0)	
deeply concerned	80 (7.7)	27 (5.6)	
Self-estimated distance to the next base station			<0.0001
Don't know	39 (2.7)	136 (22.0)	
< 500 meters	945 (56.3)	303 (49.0)	
= 500 meters	464 (32.0)	179 (29.0)	
**Adolescents**	**n = 1508**	**n = 814**	
Sex			0.009
male	730 (48.4)	440 (54.1)	
Level of education			
grammar school	743 (49.9)	253 (31.2)	<0.0001
Nationality (mother)			<0.0001
German	946 (85.1)	465 (74.9)	
Mobile phone ownership			0.004
	1373 (91.2)	711 (87.4)	
Use of a mobile phone per day			0.25
< 5 minutes	1153 (77.1)	593 (73.6)	
> 5 minutes	343 (22.9)	213 (26.5)	
Self-reported concerns about adverse health effects to EMF			<0.0001
not or little concerned	969 (87.7)	572 (92.1)	
fairly concerned	120 (10.9)	38 (6.1)	
deeply concerned	17 (1.5)	11 (1.8)	
Self-estimated distance to the next base station			<0.0001
don't know	47 (3.2)	139 (17.3)	
< 500 meters	922 (62.6)	390 (48.5)	
= 500 meters	503 (34.2)	274 (34.1)	

EMF: electromagnetic fields

### Exposure

#### Objective exposure

21 measurements hat to be excluded from the analysis due to technical errors. All exposure levels were far below the ICNIRP reference level and ranged from a mean of 0.13% (all measurement values below the limit of determination) to a mean of 0.92% of the ICNIRP reference level per second during waking hours. Median exposure was slightly higher for adolescents (0.19) than for children (0.18). The majority of measured values were below the limit of determination (82% of the measured values during waking hour).

Exposure was higher during afternoon hours than during morning hours. This applied for the children (median morning hours: 0.17, median afternoon hours: 0.19) as well for the adolescents (median morning hours: 0.18; median afternoon hours: 0.20) (table 3).

**Table 3:** Data of measured exposure to mobile telecommunication networks for the children and adolescents

**% ICNIRP-reference level**	**Children n = 1484**	**Adolescents n = 1508**
**Exposure during waking hours**		
Range	0.13–0.92	0.13–0.78
Quartiles	0.15	0.15
	0.17	0.17
	0.20	0.21
Mean (standard deviation)	0.18 (0.06)	0.19 (0.06)
**Exposure during morning**		
Range	0.13–0.80	0.13–0.74
Quartiles	0.14	0.14
	0.15	0.16
	0.19	0.20
Mean (standard deviation)	0.17 (0.06)	0.18 (0.07)
**Exposure during afternoon**		
Range	0.13–1.20	0.13–0.87
Quartiles	0.15	0.15
	0.17	0.17
	0.20	0.22
Mean (standard deviation)	0.19 (0.08)	0.20 (0.07)
**Exposure during waking hours (binary cut-off)**		
Range		
low exposure	0.13–0.25	0.25–0.92
high exposure	0.13–0.26	0.26–0.78

**Figure 2:**
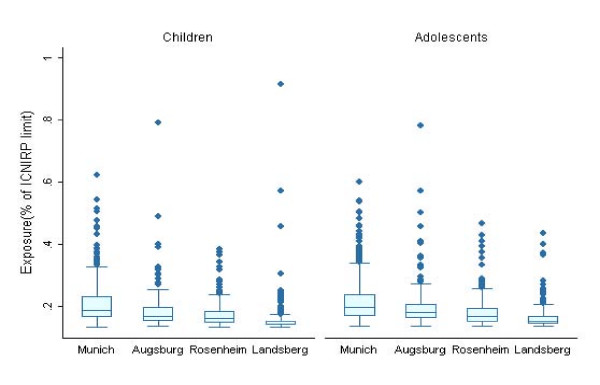
Comparison of median exposure (% ICNIRP) to mobile telecommunication networks during waking hours by study town.

Exposure varied by the size of the town of residence. Median exposure levels during waking hours were highest in Munich and lowest in the smallest town (Landsberg) (figure 2).

#### Five day measurements

Between 20% and 57% of the participants were in exactly the same exposure quartile on two days of the week (perfect agreement). Highest complete agreement was always seen between two consecutive days. However, the weekend (Friday to Saturday) differed considerably from the weekdays. For weekdays, exposure categories differed by at most one exposure quartile for more than 80% of the population indicating that misclassification of exposure might result in an underestimation of the effect (Table 4).

**Table 4:** Agreement of the exposure quartiles on different days of the week (in%)

**Weekdays**	Tuesday	Wednesday	Thursday	Friday
Monday	57 (1)/85 (2)	46/83	41/85	30/74
Tuesday		37/81	44/83	20/67
Wednesday			50/81	35/72
Thursday				31/61

(1) Perfect agreement of the exposure quartiles/(2) Deviation in at most one exposure quartile

#### Subjective exposure

Sixty-seven percent of the parents of the children and 65% of the adolescents estimated that the next mobile phone base station was less then 500 meters away from their home. Regarding the self estimated use of mobile phones during the field study, nearly every child (98%) and 90% of the adolescents stated a very low usage (< 5 minutes) in the morning hours. For the afternoon hours also 98% of the children and 86% of the adolescents reported less than 5 minutes of usage. The participating children reported longer usage of a DECT phone in the afternoon (< 5 minutes: 85%) than in the morning (< 5 minutes: 91%). The same was stated for the adolescents (afternoon: <5 minutes: 63%; morning: < 5 minutes: 86%). In general adolescents had a higher DECT phone use than children. As expected, highest use was seen in the afternoon.

## Discussion

The MobilEe-study is the first of its kind to investigate a possible association between exposure to mobile telecommunication networks and well-being in children and adolescents using personal dosimetry. In consideration of the participants' time expenditure the response of 52% was satisfying and underlines the interest in the topic of a possible association between mobile phone exposure and health.

Exposure levels were on average less than 1% of the ICNIRP reference level which is an agreement with other studies [20,21,37]. Furthermore exposure levels were highest in the largest city Munich, which is also in agreement with other studies [20,21,38].

### Strengths and limitations

The use of personal dosimetry to assess individual exposure to mobile telecommunication network frequencies enables accounting for all sources of exposure, including own and neighbour's use of mobile phones, mobile phone base stations, DECT phones and WLAN in the home environment. One major advantage of personal dosimetry is that it enables longer measurement periods and the estimation of the personal exposure not only at the place of living but also at work and during leisure activities [39,40]. Disadvantages of personal dosimetry are its low validity when the participant stays at one place for a longer time or the fact, that the body of the participant can influence the measured values [39,41,42]. Overall, personal dosimetry is considered a better measure of exposure than stationary measurements or estimation (self-reported or calculated) alone [19,40,41,43].

A drawback of the dosimeter used in this study is the limited selectivity to differentiate between the three frequency ranges. For this reason we calculated the cumulative exposure over all frequency ranges. This way we obtained a mean value which is comparable to the ICNIRP reference level. Another drawback are the measured values during night. Measuring the night-time exposure levels is a common difficulty of studies involving personal dosimetry. Our study participants placed the dosimeters near their beds, which resulted in a constant, but arbitrary measurement during the night. As shown in the lab measurements, the dosimeter measurements depend on the direction in the field. Therefore, valid measurements can only be obtained if the dosimeter is moved. It also has to be kept in mind that inside a room variations in exposure are possible if the room is e.g. close to a mobile phone base station. The position of the fixed dosimeter can lead to different measurement levels, depending on whether the dosimeter is in a wave crest or in a wave trough.

### Selection bias

Forty-five percent of the participating parents were at least fairly concerned about possible health effects of exposure to mobile phone frequencies as compared with 27% reported in a recent questionnaire study in the German general population. The proportion of concerned persons in the group of the participating adolescents was also higher (24%) than in the previous questionnaire study (14%). [44] It appears that primarily those parents and adolescents took part in the study who were concerned about a possible association between mobile phone exposure and health. We cannot rule out a preferential selection in our study of concerned subjects. Due to the objective exposure measurement a differential misclassification seems to be unlikely and therefore an overestimation of the results is also unlikely. Furthermore, several studies have shown that the Bavarian residents tend to be more concerned about mobile phone exposure and health than people living in the North or the East of Germany [20,44].

There are still missing information about non-responder as we could not reach all of these subjects for the non-response-analysis. As primarily those persons participated who were concerned about possible health effects caused by exposure to mobile telecommunication networks a selection bias is possible. Furthermore concerned participants could overestimate subjective exposure and symptoms. Due to the objective exposure assessment a differential misclassification seems to be unlikely.

### Confounding

Regarding the potential confounding variables in the association between mobile phone exposure and health it has to be kept in mind that these have to be associated with the outcome variables and exposure. While own mobile phone use might be influenced by e.g. socioeconomic status or environmental worries, it is unlikely that exposure to mobile phone base stations is largely affected. As shown in this paper adolescents and especially children's own mobile phone use was low during the study day and therefore does most likely not substantially change the overall exposure. Based on that fact, confounding is not considered a major issue in this study.

### Misclassification

Due to the fact that the measurement was limited to 24 hours, only the status quo was assessed. Yet it is possible that the individual exposure levels during the study day may not be representative. Therefore some participants carried the dosimeter for five consecutive days from Monday afternoon to Saturday afternoon. The results showed that the assessment of exposure on a single weekday reflects the typically weekday exposure quite good. However, weekend exposure differs considerably which is plausible as children and adolescents spend most parts of the weekdays in school while at weekends they might spend more time at home or at different places. Due to the fact that the change of the participants was mostly in one exposure quartile it could be that a non-differential misclassification occurred, which leads to an underestimation of the risk especially for the chronic symptoms.

In case of the subjective exposure awareness bias is possible. It could be that the participants overestimated their usage of mobile phones and DECT phones during the study day and that the self estimated distance to the next mobile phone base station was incorrect [39]. Since mainly those parents and their children respectively those adolescents participated who were concerned about possible adverse health effects to electromagnetic fields an overestimation of subjective exposure seems to be likely. Thus a differential misclassification and overestimation of the results is possible which might result in specious findings.

The examined outcomes were also assessed once. Therefore a misclassification concerning chronic symptoms is also possible. It might be that the participants overestimated their symptoms during the measurement day, because they were more aware of them. As the participants were not aware of their actual objective exposure level differential misclassification is unlikely [45-47].

### Statistical methods

Due to the results of the laboratory measurements we decided to replace all values that were below the limit of determination (0.05 V/m) by half of this limit instead of taking the real measured values. One has to keep in mind that this could lead to more conservative results. In a pilot study we used different methods for the handling of values below the limit of determination like e.g. multiple imputation for the children's data. No differences in the calculated Odds Ratios and the Confidence Intervals were seen, therefore our way of substitution of the values below the limit of determination is not considered to bias the results [48].

We a priori decided to analyse the association between measured objective exposure and well-being using exposure levels as quartiles. This enabled us to detect possible dose-response relationships. We could not take exposure as a continuous variable, because too many values were below the limit of determination.

In a sensitivity analysis we divided exposure at 90% percentile to compare those 10% of the participants who had the highest exposure levels to the remaining participants. One reason to use also a binary cut-off has been that the range of exposures within the highest quartile seemed to be larger than differences between quartiles. In another sensitivity analysis we also evaluated exposure separately for the three frequency bands. Due to the different models in the analysis, multiple comparisons have to be taken into account.

## Conclusion

In summary, this study is the first to use personal dosimetry to assess the individual exposure to mobile telecommunication networks in children and adolescents, enabling objective assessment of exposure from all sources. Participation in the field study was fairly good and dosimeters were well accepted. We found an exposure to mobile phone frequencies far below the current ICNIRP reference levels.

## Abbreviations

CAPI: Computer Assisted Personal Interview; DECT: Digital Enhanced Cordless Phone; EMF: electromagnetic fields; GSM: Global System for Mobile Communications; ICNIRP: International Commission on Non-Ionizing Radiation Protection; UMTS: Universal Mobile Telecommunication System; WLAN: Wireless Local Area Network

## Competing interests

The authors declare they have no competing financial interests.

## Authors' contributions

ST was the one of the principle investigators responsible for design, conduct and writing the manuscript. AK was also one of the principle investigators and responsible for data analysis and interpretation of the data. SH was also one of the principle investigators responsible for design and acquisition of data. GP coordinated the technical parts of the study. RK made contributions to draft the manuscript. KR made contributions to conception and design and also to analysis and drafting the manuscript. All authors read and approved the final manuscript.
